# Clinical management of gastric cancer: results of a multicentre survey

**DOI:** 10.1186/1471-2407-11-369

**Published:** 2011-08-24

**Authors:** Xiaolong Zhang, Nanjing Li, Wen Wei, Wenxiu Yao, Ke Xie, Jiankun Hu, Lida Shen, Weizheng Ji, You Lu, Feng Wen, Yu Jiang, Feng Xu, Hong Feng, Feng Bi, Qiu Li

**Affiliations:** 1 The Department of Medical Oncology, Cancer Center, State Key Laboratory of Biotherapy, West China Hospital, Sichuan University; No. 37, Guo Xue Xiang, Chengdu, Sichuan, 610041, China; 2 The Department of Medical Oncology, Sichuan Cancer Hospital; No.55, Ren Min Nan Road Si Duan, Chengdu, Sichuan, 610041, China; 3 Oncology Department, Sichuan Academy of Medical Sciences & Sichuan Provincial People's Hospital; No.32, First Ring Road Xi Er Duan, Chengdu, Sichuan, 610041, China; 4 The Department of Gastrointestinal Surgery, West China Hospital, Sichuan University; No. 37, Guo Xue Xiang, Chengdu, Sichuan, 610041, China; 5 The Department of Medical Oncology, Yunnan Cancer Hospital; Ren Min Xi Road, Kunming, Yunnan, 650001, China; 6 The Department of Medical Oncology, Affiliated Hospital of Xinjiang Medical College; No.1, Liyushan Road, Urumqi, Xiangjiang, 830054, China; 7 The Department of Radiation Oncology, Cancer Center, State Key Laboratory of Biotherapy, West China Hospital, Sichuan University; No. 37, Guo Xue Xiang, Chengdu, Sichuan, 610041, China; 8 West China School of Public Health, Sichuan University, No.17 Section 3, Renmin South Road, Chengdu, Sichuan, 610044, China

## Abstract

**Background:**

The National Comprehensive Cancer Network clinical practice guidelines in oncology-gastric cancer guidelines have been widely used to provide appropriate recommendations for the treatment of patients with gastric cancer. The aim of this study was to examine the adherence of surgical oncologists, medical oncologists, and radiation oncologists' to the recommended guidelines.

**Methods:**

A questionnaire asking the treatment options for gastric cancer cases was sent to 394 Chinese oncology specialists, including surgical oncologists, medical oncologists, and radiation oncologists working in hospitals joined in The Western Cooperative Gastrointestinal Oncology Group of China. The questionnaire involved a series of clinical scenarios regarding the interpretation of surgery, neoadjuvant, adjuvant, and advanced treatment planning of gastric cancer.

**Results:**

Analysis of 358 respondents (91%) showed variations between each specialization and from the recommended guidelines in the management approaches to specific clinical scenarios. The majority of specialists admitted that less than 50% of patients received multidisciplinary evaluation before treatment. The participants gave different responses to questions involving adjuvant, neoadjuvant, and advanced settings, compared to the recommended guidelines.

**Conclusions:**

These results highlight the heterogeneity of the treatment of gastric cancer. Surgical oncologists, medical oncologists, and radiation oncologists are not adhering to the recommended guidelines.

## Background

Despite the downward trend for cancer incidence and mortality in most countries, gastric cancer has become the second leading cause of cancer-related deaths worldwide with an annual rate of 700,000 deaths [1]. Almost two-thirds of the cases occur in developing countries, with 42% occurring in China alone, where gastric cancer remains the most deadly cancer among both sexes [2].

Surgical resection, chemotherapy and radiotherapy are mainstay of treatment for patients with gastric cancer [3-5]. However, several questions concerning the treatment of gastric cancer remain. First, the type of resection and the role of extensive lymphadenectomy have been the subjects of international debate. Second, is there an ideal setting or regimen that predicts whether chemotherapy and/or radiotherapy should be provided before and/or after surgery as (neo) adjuvant treatment in patients with localized disease? Finally, there is no internationally accepted standard of care for patients with metastatic and advanced cancer, and uncertainty remains regarding the choice of the regimens [6,7].

Clinical practice guidelines in oncology were developed to ensure quality cancer care throughout the world. Several global organizations have developed clinical practice guidelines for the treatment and management of gastric cancer. These groups include the National Comprehensive Cancer Network (NCCN) [8], the European Society of Clinical Organization [9], and the Japanese Gastric Cancer Association [10]. The NCCN gastric cancer guidelines (the Guidelines) have been widely used in China to reduce any disparities in treatment between different institutions for the treatment of patients with gastric cancer. However, we found in multidisciplinary team specialists from different department usually give different recommendations for surgery, neoadjuvant, adjuvant, and advanced treatment planning for patients. Until now no studies have been carried out in regard to variations of the clinical management of gastric cancer. Accordingly, the aim of this study was to examine the adherence of surgical oncologists, medical oncologists, and radiation oncologists' to the Guidelines.

## Methods

### Survey development

The inclusion criterions were as follows: (1) All participants were oncology specialists, which included surgical oncologists, medical oncologists, and radiation oncologists who mainly treat gastric cancer patients. (2) Subjects must have worked in an oncology specialist field for at least five years; and (3) Subjects should be well enough to provide informed consent and finish the questionnaire. Criteria for exclusion were (1) subjects with gastric cancer themselves or (2) participants who did not complete their questionnaire for any reason. Before administration, the questionnaire was pilot tested using 20 oncology clinicians, and then revised for clarity and ease of comprehension. The questionnaire contained a total of 32 questions. The first five questions were related to the oncologists and their hospital characteristics, including demographic information (gender, age), and their training and educational backgrounds. The participants were asked "What percentage of your patients undergoes a multidisciplinary evaluation before treatment?" Then, individual items were constructed to measure participants' adherence to the Guidelines in medical practice. Questions 7 to 11 were related to surgical options, including the type of resection (total gastrectomy or subtotal gastrectomy), the adequate margin in complete resection, and the extent of lymph node dissection. There were three additional questions asking about the follow-up of patients after complete resection. The questionnaire then presented a number of clinical scenarios and elicited the appropriate treatment recommendations using various forms of adjuvant therapy, neoadjuvant therapy, and advanced options, respectively.

### Data collection

The study was approved by the institutional review board of the West China Hospital, Sichuan University, China. Questionnaires were handed out to all eligible participants working in hospitals joined in The Western Cooperative Gastrointestinal Oncology Group of China, a cooperative group consortium of affiliated hospitals of universities and large medical centers in the west of China.

Questionnaires were handed out to all eligible participants at the same time at each site. All of them were given information explaining the study and asked not to share their answers with their colleagues. Participants themselves without signing should complete questionnaires to ensure that investigators did not know their name when the data were analyzed. Participants were asked to finish and hand in the survey on the spot. All answers were entered into a computerized database by two independent people. Then the data were analyzed by an independent investigator, a third person who had nothing to do with the data collection. The survey is attached as additional file 1 to this paper.

### Statistical analysis

Comparisons between answers from surgical oncologists, medical oncologists, and radiation oncologists respondents are mainly based on Chi-square, and Wilcoxon rank sum test as appropriate. Chi-square test is performed for the categorical data, and Wilcoxon rank sum test is used for the ordinal data [11]. The significance level was set at P < 0.05. SPSS statistic software (Version 13.0) was used for data analysis.

## Results

### Characteristics of oncology specialists and centers

Three hundred ninety-four oncology clinicians were initially invited to participate in this study. Three hundred sixty-three responses were received, of which 358 were useable (response rate: 91%). Five (1.27%) participants responded but did not complete their questionnaires. Thirty-one (7.87%) oncology specialists did not hand in their questionnaire. The median age of the participants was 40 years (range 29-68 years). The study consisted of 161 (44.97%) medical oncologists, 101 surgical oncologists, and 96 radiation oncologists. About half of the participants had worked in an oncology specialty for more than 15 years. There were more participants from university hospitals. The characteristics of the participants and centers are summarized in Table 1.

**Table 1:** Oncology Specialists and Center Characteristics

	Respondents	
Characteristics	N	%
Number of respondents	358	90.86
Sex		
Male	206	57.54
Female	152	42.46
Specialty		
Medical oncologists	161	44.97
Oncological surgeons	101	28.21
Radiation oncologists	96	26.82
Number of years as an oncology clinician		
15 years or less	177	49.44
More than 15 years	181	50.56
Practice setting		
General hospital	126	35.20
University hospital	232	64.80

### Practice variation in clinic

#### Multidisciplinary evaluation

The participants were asked to estimate what percentages of their patients undergo multidisciplinary evaluation before treatment. The majority reported that less than half of all patients received multidisciplinary evaluation before treatment. Only 10 (2.79%) and 26 (7.26%) of participants reported that 50-75% or > 75% of their patients undergo multidisciplinary evaluation before treatment, respectively. Furthermore, we found there was no difference in this question between participants from university hospitals and those from general hospitals (*P *= 0.325). However, participants with a short time in practice (< 15 y) were more likely to give their patients multidisciplinary evaluation (*P *= 0.009).

#### Principles of surgical treatment

When it came to selecting the type of surgery, almost all the surgical oncologists selected subtotal gastrectomy for the clinical scenario involving a patient with distal gastric cancer. Most surgical oncologists (76.24%, n = 77) thought that 5 cm was an adequate margin for complete resection of gastric cancer, while D2 dissection was considered an essential procedure by 89 (88.12%) surgical oncologists. Seventy-three (72.28%) thought at least 15 lymph nodes should be removed. These responses were consistent with the Guidelines [8]. However, the surgical procedure of choice for proximal gastric cancer was more controversial, as mentioned in the Guidelines [8]. Interestingly, the responses of the medical oncologists and radiation oncologists vary greatly with regards to the type of gastrectomy and the extent of the lymph node dissection. Table 2 summarizes the choices for the clinical scenarios related to surgery posed to the oncology specialists.

**Table 2:** Comparison of surgery practice

Surgery practice	Recommendation of the Guidelines	Oncological surgeons		Medical oncologists		Radiation oncologists		*p*
			
		N	%	N	%	N	%	
proximal cancer cT2N0M0	Subtotal gastrectomy							0.329
Total gastrectomy		34	33.66	69	42.86	37	38.54	
Subtotal gastrectomy		67	66.33	92	57.14	59	61.46	
antrum cancer cT2N0M0	Controversial: total or subtotal gastrectomy							< 0.001
Total gastrectomy		4	3.96	36	22.36	33	34.38	
Subtotal gastrectomy		97	96.04	125	79.64	63	65.63	
Adequate margin	5 cm							< 0.001
2 cm		0	0	4	2.48	3	3.13	
3 cm		8	7.92	19	11.80	8	8.33	
4 cm		14	13.86	41	25.47	23	23.96	
5 cm		77	76.24	58	36.02	33	34.38	
6 cm		2	1.98	17	10.56	14	14.58	
unclear		0	0	22	13.66	15	15.63	
Extent of lymph nodes dissection	D2							0.748
D1		0	0	14	8.70	8	8.33	
D2		89	88.12	94	58.39	61	63.54	
Greater than D2		12	11.88	37	22.98	15	15.63	
unclear		0	0	16	9.94	12	12.50	
at least how many regional lymph nodes should be removed	15							0.041
12		0	0	11	6.83	0	0	
15		73	72.28	68	42.24	44	45.83	
20		19	18.81	46	28.57	39	40.63	
30		9	8.91	13	8.07	0	0	
unclear		0	0	23	14.29	13	13.54	

#### Principles of neoadjuvant treatment

Table 3 represents the data regarding options for neoadjuvant treatment. The Guidelines recommend neoadjuvant chemotherapy for patients with stage T2-4, or N+ [8]. Just under half (47.52%) of the oncological surgeons selected neoadjuvant chemotherapy and 12.87% selected preoperative chemoradiation for the clinical scenario of a patient with stage cT4N0M0. Similarly, some surgical oncologists selected neoadjuvant chemotherapy (17.82%) or chemoradiation (12.87%) for a patient with local lymph node metastasis. None of the participants selected radiotherapy alone as neoadjuvant treatment. Preference for a chemotherapy regimen varied greatly. There was very little difference in the responses regarding the duration of neoadjuvant treatment among medical oncologists, radiation oncologists, and surgical oncologists (*P *= 0.21), and this is consistent with the Guidelines [8].

**Table 3:** Medical decision making on neoadjuvant treatment

Neoadjuvant practice	Recommendation of the Guidelines	Oncological surgeons		Medical oncologists		Radiation oncologists		*p*
			
		N	%	N	%	N	%	
adenocarcinoma cT4N0M0	gastrectomy or preoperative chemotherapy							0.612
gastrectomy		40	39.60	75	46.58	27	28.13	
preoperative chemotherapy		48	47.52	72	44.72	41	42.71	
preoperative radiotherapy		0	0	0	0	0	0	
preoperative chemoradiation		13	12.87	14	8.70	28	29.17	
adenocarcinoma cT2N2M0	gastrectomy or preoperative chemotherapy							0.040
gastrectomy		70	69.31	77	47.83	30	31.25	
preoperative chemotherapy		18	17.82	73	45.34	38	39.58	
preoperative radiotherapy		0	0	0	0	0	0	
preoperative chemoradiation		13	12.87	11	6.83	28	29.17	
regimen usually used in neoadjuvant setting	ECF(EPI+DDP+5FU)							0.634
CF(DDP+5FU)		21	20.79	29	18.01	15	15.63	
ECF(EPI+DDP+5FU)		15	14.85	35	21.74	14	14.58	
fluoropyrimidine(5Fu or Capecitabine)		24	23.76	7	4.35	7	7.30	
oxaliplatin based regimen		23	22.77	63	39.13	35	36.46	
irinotecan based regimen		12	11.88	5	3.11	9	9.38	
paclitaxel or docetaxel based regimen		6	5.94	22	13.66	16	16.67	
combination of above or other		0	0	0	0	0	0	
how long neoadjuvant chemotherapy last	3 cycles, about 2 months							0.213
< 1 m		21	20.79	19	11.80	8	8.33	
1-3 m		76	75.25	127	78.88	79	82.29	
> 3 m		4	3.96	15	9.32	9	9.38	

#### Principles of adjuvant treatment

The Guidelines recommend adjuvant chemoradiation for patients with stages T3-4, or N+, after R0 resection [8]. All the medical oncologists and radiation oncologists recommended adjuvant treatment for the clinical scenario involving a patient with stage pT4N0M0, while 18% of surgical oncologists chose to observe the patient instead of adjuvant treatment. Moreover, there were differences between medical oncologists and radiation oncologists in terms of adjuvant treatment options. For those patients staged as T2N0M0 with high risk features, such as poorly differentiated or higher grade cancer, lymphovascular invasion, or < 50 years of age, the Guidelines recommend observation, adjuvant chemotherapy, or adjuvant chemo-radiation [8]. Surgical oncologists appeared to pay more attention to the extent of the lymph node dissection, with approximately 70% stating that they would give adjuvant treatment to a patient who has only six lymph nodes excised. In contrast, medical oncologists and radiation oncologists were more likely to give adjuvant treatment to patients with poorly differentiated and lymphovascular invasion. However, they were also more likely to observe a patient who has an inadequate lymph node dissection without adjuvant treatment. The participants were highly consistent on the start time and duration of adjuvant chemotherapy, which the Guidelines do address [8]. However, the preference of which chemotherapy regimen varies greatly. Table 4 summarizes the data regarding the options for adjuvant treatment after complete resection.

**Table 4:** Medical decision making on adjuvant treatment

Adjuvant practice	Recommendation of the Guidelines	Oncological surgeons		Medical oncologists		Radiation oncologists		*p*
			
		N	%	N	%	N	%	
moderately differentiated adenocarcinoma pT4N0M0 after gastrectomy	postoperative chemoradiation							0.007
observe without postoperative treatment		19	18.81	0	0	0	0	
postoperative chemotherapy		57	56.44	102	63.35	26	27.08	
postoperative radiotherapy		0	0	0	0	7	7.29	
postoperative chemoradiation		25	24.75	59	36.65	63	65.63	
moderately differentiated adenocarcinoma pT4N0M0 after gastrectomy	Systemic chemotherpy							< 0.001
Systemic chemotherapy		73	72.28	89	55.28	75	78.13	
Intraperitoneal chemotherapy(IP)		0	0	0	0	0	0	
Systemic chemotherapy+IP		28	27.72	72	44.72	21	21.88	
Other		0	0	0	0	0	0	
moderately differentiated adenocarcinoma invaded muscularis and vascular without metastasis in the 16 removed regional lymph nodes	postoperative chemotherapy							0.044
observe without postoperative treatment		41	40.59	37	22.98	26	27.08	
postoperative chemotherapy		46	45.54	73	45.34	38	39.58	
postoperative radiotherapy		0	0	0	0	13	13.54	
postoperative chemoradiation		14	13.86	51	31.68	19	19.79	
other		0	0	0	0	0	0	
poor differentiated adenocarcinoma invaded muscularis without metastasis in the 16 removed regional lymph nodes	postoperative chemotherapy							0.053
observe without postoperative treatment		52	51.49	21	13.04	17	17.71	
postoperative chemotherapy		43	42.57	140	86.96	58	60.42	
postoperative radiotherapy		3	2.97	0	0	0	0	
postoperative chemoradiation		3	2.97	0	0	21	21.88	
other		0	0	0	0	0	0	
well differentiated adenocarcinoma invaded muscularis without metastasis in the 6 removed regional lymph nodes	observe without postoperative treatment							0.020
observe without postoperative treatment		29	28.71	68	42.24	36	37.50	
postoperative chemotherapy		66	65.35	61	37.89	36	37.50	
postoperative radiotherapy		0	0	0	0	5	5.21	
postoperative chemoradiation		6	5.94	32	19.88	19	19.79	
other		0	0	0	0	0	0	
when begin adjuvant chemotherapy after gastrectomy	NA							0.233
1-2 w		13	12.87	28	17.39	11	11.46	
3-4 w		82	81.19	133	82.61	79	82.29	
5-6 w		4	3.96	0	0	6	6.25	
≥ 6 w		2	1.98	0	0	0	0	
regimen usually used in adjuvant setting (without neoadjuvant chemotherapy)	CF(DDP+5FU) or fluoropyrimidine(5Fu or Capecitabine)							0.383
CF(DDP+5FU)		18	17.82	21	13.04	12	12.50	
ECF(EPI+DDP+5FU)		12	11.88	25	15.53	7	7.29	
fluoropyrimidine(5Fu or Capecitabine)		12	11.88	11	6.83	11	11.46	
oxaliplatin based regimen		34	33.66	69	42.86	44	45.83	
irinotecan based regimen		20	19.80	8	4.97	9	9.38	
paclitaxel or docetaxel based regimen		5	4.95	24	14.91	8	8.33	
combination of above or other		0	0	3	1.86	5	5.21	
how long adjuvant chemtherapy last	NA							0.220
1-3 m		11	10.89	2	1.24	11	11.46	
4-6 m		71	70.30	144	89.44	63	65.63	
7-9 m		7	6.93	3	1.86	7	7.29	
10-12 m		5	4.95	5	3.11	8	8.33	
≥ 12 m		3	2.97	4	2.48	7	7.29	
3-6 m in the first year, then 2-3 m annually for 2-3 y		4	3.96	3	1.86	0	0	
other		0	0	0	0	0	0	

#### Principles of follow-up

Table 5 represents the responses to questions about follow-up. There appeared to be a uniform consensus among participants on the question concerning follow-up, but the answers were different from the Guidelines [8], to some extent. More than half of the participants recommended a patient undergo follow-up every 2-3 months for the first 3 years, then once every year, while the Guidelines recommend every 4-6 months for the first 3 years, then annually [8]. All the participants in this investigation stated that they would undertake a complete medical history, physical examination, and complete blood count. The majority reported that they would test the patient's hepatic and renal function, and endoscopy. All the oncological surgeons selected abdominal CT scan, while less than half would ask for a chest CT scan. In contrast, medical oncologists and radiation oncologists tended to select chest CT scan as well as an abdominal CT scan. However, only 17-28% of participants would assess a patient's Vitamin B12 and trace elements along with electrolytes. The majority of participants would recommend that patients take supplements such as iron, vitamin B12, and trace elements.

**Table 5:** Medical decision making on follow-up

Follow-up practice	Recommendation of the Guidelines	Oncological surgeons		Medical oncologists		Radiation oncologists		*p*
			
		N	%	N	%	N	%	
how often follow-up	Every 4-6 m for 3 y, then annually							0.086
Every 4-6 m for 3 y, then annually		9	8.91	18	11.18	7	7.29	
Every 4-6 m for 5 y, then annually		13	12.87	25	15.53	11	11.46	
Every 2-3 m for 3 y, then annually		64	63.37	94	58.39	63	65.63	
Every 2-3 m for 5 y, then annually		12	11.88	19	11.80	14	14.58	
other		3	2.97	5	3.11	1	3.13	
which included in the follow-up (multiple answers)	Complete history and physical examination for all the patients, CBC, platetets, radiologic imaging or endoscopy as clinically indicated, monitor Vit B12 for proximal or total gastrectomy patitnts							0.371
Complete history and physical examination		101	100	161	100	96	100	
CBC, Hb, platetets		101	100	161	100	96	100	
Hepatic and renal function		101	100	154	95.65	91	94.79	
Endoscopy		92	91.09	123	76.40	86	89.58	
Chest CT		46	45.54	156	96.89	88	91.67	
Abdominal CT		101	100	160	99.38	93	96.88	
Vit B12		29	28.71	34	21.12	26	27.08	
Trace elements and electrolytes		18	17.82	29	18.01	19	19.79	
Other		0	0	0	0	0	0	
which recommended as food supplementation (multiple answers)?	Vit B12 for proximal or total gastrectomy patitnts							0.786
Iron		101	100	159	98.76	82	85.42	
Trace elements and electrolytes		71	70.30	128	79.50	74	77.08	
Vit B12		101	100	161	100	95	98.96	
Other		0	0	0	0	0	0	

#### Principles of palliative treatment

More than 70% of the participants in our study recommended a liver resection and systemic chemotherapy for a patient presented with a solitary liver metastases. It is interesting that almost all the participants agreed to give intraperitoneal chemotherapy and systemic chemotherapy for an advanced gastric cancer patient with large ascites, although the Guidelines do not address intraperitoneal chemotherapy. The Guidelines recommend taxanes based regimens or ECF regimen with category 1 recommendation [8]. When asked, "What was their choice as the first line chemotherapy regimen?", the first three choices for first line chemotherapy by surgical oncologists and medical oncologists were the same: oxaliplatin based regimen, paclitaxel or docetaxel based regimen, and ECF. On the other hand, radiation oncologists selected the paclitaxel or docetaxel based regimen, oxaliplatin based regimen, and irinotecan based regimen. There is no established second, or third-line therapy according to the Guidelines [8]. The majority of the participants in this study would recommend the second or third line chemotherapy when the patient's performance status (PS) is good. The percentage of participants who tend to give best supportive care instead of chemotherapy increases for a patient with worse PS. Table 6 summarizes the responses to questions about treatment of patients with advanced gastric cancer.

**Table 6:** Medical decision making on advanced treatment

Advanced practice	Recommendation of the Guidelines	Oncological surgeons		Medical oncologists		Radiation oncologists		*p*
			
		N	%	N	%	N	%	
adenocarcinoma with solitary liver metastases	Systemic chemotherpy							0.443
Systemic chemotherpy		4	3.96	7	4.35	5	5.21	
Liver lesion resection		8	7.92	16	9.94	5	5.21	
TACE		10	9.90	21	13.04	15	15.63	
Liver lesion resection + systemic chemotherpy		79	78.22	117	72.67	68	70.83	
Other		0	0	0	0	3	3.13	
adenocarcinoma with large quantity of ascites	Systemic chemotherpy							0.210
Systemic chemotherapy		3	2.97	2	1.24	2	2.08	
intraperitoneal chemotherapy(IP)		4	3.96	6	3.73	5	5.21	
Systemic chemotherapy+IP		94	93.07	153	95.03	89	92.71	0.254
Other		0	0	0	0	0	0	
lungs metastases	Paclitaxel or docetaxel based regime or ECF(EPI+DDP+5FU)							0.070
CF(DDP+5FU)		2	1.98	3	1.86	1	1.04	
ECF(EPI+DDP+5FU)		17	16.83	23	14.29	9	9.38	
Fluoropyrimidine(5Fu or Capecitabine)		13	12.87	12	7.45	14	14.58	
Oxaliplatin based regimen		33	32.67	51	31.68	25	26.04	
Irinotecan based regimen		9	8.91	22	13.66	15	15.63	
Paclitaxel or docetaxel based regime		25	24.75	46	28.57	29	30.21	
Combination of above or other		2	1.98	4	2.48	3	3.13	
lungs metastases, failure after ECF(EPI+DDP+5FU)	Change regimen and continue chemotherapy							
Change regimen and continue chemotherapy		101	100	156	96.89	83	86.46	0.137
Best supportive care		0	0	2	1.24	9	9.38	
Other		0	0	3	1.86	4	4.17	
lungs metastases, failure after both ECF and Paclitaxel+DDP	Change regimen and continue chemotherapy							0.351
Change regimen and continue chemotherapy		86	85.15	122	75.78	60	62.50	
Best supportive care		13	12.87	35	21.74	31	32.29	
Other		2	1.98	4	2.48	5	5.21	
lungs metastases, failure after both ECF and Paclitaxel+DDP, PS 3	Best supportive care							0.222
Change regimen and continue chemotherapy		18	17.82	15	9.32	9	9.38	
Best supportive care		79	78.22	136	84.47	81	84.38	
Other		4	3.96	10	6.21	6	6.25	

## Discussion

Quality cancer care depends on research findings, treatment improvements, and practice guidelines. Clinical guidelines have been designed to maximize the effectiveness of care as an important tool in evidence based practice, may reduce inappropriate variations in treatment approaches, and allow for continuous monitoring of appropriate care delivery. However, the practice guidelines are wasted unless they are used in everyday practice by clinical oncologists [12-14]. In this study, we found considerable variation when comparing the recommendations of the Guidelines and routine practice of different oncology specialists.

In recent years, a series of studies in various cancers demonstrated that multidisciplinary care could achieve greater resource efficiency and improve standards of care through a reduction in duplication and gaps in service provision, enabling the delivery of holistic services and better continuity of care [15]. Effective multidisciplinary team functioning is important not only for the benefit of patients, but also for the efficiency, morale, and work satisfaction of the individual team members [16]. Multidisciplinary care has become the standard in cancer management, which is supported by national and international clinical practice guidelines in many countries [17]. The management of gastric cancer usually requires the expertise of several disciplines, including surgical oncology, radiation oncology, medical oncology, nutritional support, and endoscopic expertise. The Guidelines encourage a multidisciplinary evaluation for the treatment of patients with gastric cancer [8]. However, approximately 90% of the participants of our survey reported that less than half of all patients received multidisciplinary evaluation before treatment. Through stratification analysis, we found that there was no difference between participants from university hospitals and those from general hospitals, but participants with a short time in practice (5-15 y) were more likely to have their patients to receive multidisciplinary evaluation. Perhaps experienced clinicians are more confident in making treatment decisions alone.

The type of resection and the role of extensive lymphadenectomy have been the subject of debate according to the Guidelines [8]. In this study, D2 dissection was generally considered as an essential procedure, however, surgical oncologists have different ideas as to the surgical procedure of choice for gastric cancer, such as the gastrectomy options for proximal gastric cancer, the extent of the lymph nodes dissection, and what is an adequate surgical margin. There are more controversial issues with regards to the treatment principles among medical oncologists and radiation oncologists, with many of them unfamiliar with the principals of surgery. In addition, the participants provided different selections for questions on the adjuvant, neoadjuvant, and advanced settings, compared to the Guidelines.

A number of factors are considered during the process of formulating a treatment plan for gastric cancer patients: Most importantly, how well a clinician adheres to the practice guidelines depends largely on the strength of the evidence supporting clinical practice. If a clinician finds that the evidence is scant or if the evidence is not suitable for their patients, then they could not adhere to the guidelines. We have found evidence to support this issue in this study. For example, the role of adjuvant therapy for gastric cancer has been controversial given the lack of high level, worldwide evidence. Although numerous phase III studies, including a surgery-only group, have been reported in the last three decades, definitive evidence of the efficacy of postoperative chemotherapy is lacking [18]. The first positive, large scale phase III trial by the Adjuvant Chemotherapy Trial of S-1 for Gastric Cancer (ACTS-GC) group reported the superiority of S-1 as an adjuvant chemotherapy over surgery alone after D2 lymph node dissection in a Japanese population [19]. The effect of adjuvant chemotherapy outside of East Asia is uncertain, and standard management following curative surgery is heterogeneous throughout the world [18]. Recent meta-analyses have suggested a small benefit of adjuvant therapy, but the widely varying regimens and populations from the studies included in these meta-analyses have limited their impact [6,7,18,20,21]. In addition, the landmark trial in chemoradiation cited in the Guidelines was the intergroup trial INT-0116 [22]. However, 90% of the patients enrolled in this study received D0 (54%) or D1 (36%) dissection, while D2 dissection is widely performed in China. It is estimated that the radiation in INT-0116 makes up for the inadequacy of surgery. Again, intraperitoneal chemotherapy was widely accepted by the participants in this study while it was not addressed in the Guidelines at all. This may be explained by the geographical variation in standard practice, which really exists. For instance, adjuvant oral fluoropyrimidine monotherapy following radical D2 resection is the standard care of stage II-III gastric cancer in Japan. In Europe, combination chemotherapy delivered before and after D2 resection is the most widely accepted practice, and in the USA, patients will routinely be referred for adjuvant chemoradiation following resection [23]. There is no worldwide, high-level evidence on the treatment of gastric cancer, hence, it can be expected that clinicians do not adhere to the Guidelines. Secondly, some of this variation appears to represent professional preferences. Recent studies have shown that perioperative chemotherapy yields a substantial pathologic response that result in durable survival time [24]. The British Medical Research Council performed the first well-powered phase III trial (MAGIC trial) for perioperative chemotherapy [25]. The result has established perioperative chemotherapy as an option for the standard of care of patients with resectable gastric cancer, according to the Guidelines [8]. In our study, there were few surgical oncologists who preferred neoadjuvant chemotherapy. Finally, a lack of consistency among clinicians may also be the result of recognition error and unfamiliarity with the use of accepted criteria. In this study, a few medical oncologists and radiation oncologists were not clear about the surgical principle behind treating gastric cancer. In addition, the rapid dissemination of new knowledge makes it more difficult for clinicians to keep up to date, and inconsistent reports in the literature can also contribute to variability in treatment decision making among clinicians.

We acknowledge there are several limitations to the current analysis. Firstly, we asked participants to answer based on what they "usually do with most" of their patients. However, respondents were more likely to select "one best answer". Their answer may be different from their actual decision-making in the oncology clinic. The second limitation is that we did not elaborate further on many of our findings due to our concern about the length of the survey and ensuring responders' anonymity. For example, we did not collect data about institutional affiliations to explore whether practice was consistent within the same division. Thirdly, we did not perform multivariate analyses for each clinical scenario to analyze differences according to the type of practice (university vs. other), age, gender, experiences of participants, and volume of a center, etc. However, these factors could also affect the clinician's practice. For example, it was reported that surgeons who do more gastric resections per year were more likely to report performing a D2 resection, according to a study in Canada [26]. In addition, it is unclear whether the area of specialty (surgery vs. medical) affects the treatment option. We could not determine from the current study how a surgeon affects the choices of a chemotherapy regimen or how a medical oncologist affects the type of resection. Finally, and perhaps most importantly, several of the scenarios chosen were purposefully controversial without clear evidence to suggest one optimal approach.

Nevertheless, we believe that the practice variation documented here should encourage the planning and implementation of randomized clinical trials to address controversial areas of the treatment of gastric cancer. The significant increase in the quantity of scientific clinical trials should result in guidelines updated with more certainty and supporting evidence. This survey should also encourage specialists in the field of gastric cancer to keep up to date with new knowledge with a focus on the use of accepted treatment criteria. More continuing medical education programs should be designed to shorten the knowledge gap and improve the quality of care and outcome of multidisciplinary management. The documentation of practice variation highlights areas where better education, dissemination of information, and quality improvement systems can improve care, as long as the evidence for a practice has also increased with additional studies.

## Conclusions

These results highlight the heterogeneity of the treatment of gastric cancer. Surgical oncologists, medical oncologists, and radiation oncologists are not adhering to the recommended guidelines. The study should encourage the planning and implementation of randomized clinical trials to address controversial areas of the treatment of gastric cancer and to establish updated guidelines with more certainty and supporting evidence. It should also encourage the development of more continuing medical education programs designed to shorten the knowledge gap and to improve the quality of care and outcome of multidisciplinary management. Specialists in the field of gastric cancer should keep up to date with new knowledge with a focus on the use of accepted treatment criteria.

## Competing interests

The authors declare that they have no competing interests.

## Authors' contributions

XZ designed the study and drafted the manuscript. WY, KX, JH, LS, WJ, and YL was responsible for data acquisition. NL and WW were responsible for quality control of data and algorithms. FW was responsible for data analysis and interpretation. YJ, FX and HF were responsible for statistical analysis. FB critically reviewed the manuscript. QL participated in the overall design, study coordination and finalized the draft of the manuscript. All authors read and approved the final manuscript.

## Pre-publication history

The pre-publication history for this paper can be accessed here:

http://www.biomedcentral.com/1471-2407/11/369/prepub

## Supplementary Material

Additional file 1 **Gastric cancer treatment survey**. questionnaires used in this study. Click here for file
