# Supplementary material for: Clinical management of gastric cancer: results of a multicentre survey
Source: BMC Cancer. 2011 Aug 24;11:369. doi: 10.1186/1471-2407-11-369 (PMC3224384; doi:10.1186/1471-2407-11-369)
Supplement: Additional file 1 — Gastric cancer treatment survey. questionnaires used in this study. [file 1471-2407-11-369-S1.DOC]

Dear Doctor,

We are conducting a survey of doctors regarding practice differences in the treatment of gastric cancer. The survey was approved by the medical ethics committee of West China Hospital, Sichuan University in China.

The survey consists of a number of questions about how you manage common issues in the treatment of gastric cancer. As you will notice, there are no right or wrong answers. We are interested in your routine practice, that is, what you usually do for your average patient. We will keep your answers confidential and combine them with those of other doctors for analysis. No identifying information will be reported. All survey answers are encrypted.

We understand your time is extremely valuable. We have tried to make this survey concise and useful. We estimate it will take you approximately 10 min to complete the survey.

Thank you very much!

Sincerely yours,

Qiu Li, M.D., Ph.D.

1. You are: A. male B. female
2. You are: A. medical oncologist B. radiologist C. surgeon
3. Your age is _________
4. How many years have you worked as oncology clinician?
5. 5-15 years B. More than 15 years

5. Your practice setting?

A. general hospital B. university hospital

6. How many patients undergo multidisciplinary evaluation before treatment?

A. <25% B. 25-50% C. 51-75% D.76-100%

7. A 45y otherwise healthy male with proximal lesser curvature cancer cT2N0M0. What would you recommend? A. Total gastrectomy B. Subtotal gastrectomy

8. A 47y otherwise healthy female with distal (antrum)gastric cancer cT2N0M0. What would you recommend? A. Total gastrectomy B. Subtotal gastrectomy

1. How long is the adequate margin in complete resection?

A. 2cm B.3cm C.4cm D.5cm E.6cm. F.unclear

10. Which should bedone in gastric resection when it comes to lymph nodes dissection in your opinion?

A. D1 B. D2 C. greater than D2 D. unclear

11. How many regional lymph nodes at least should be removed in gastric resection in your opinion?

A.12 B.15 C.20 D.30 E. unclear

12. A 61y otherwise healthy male with gastric adenocarcinoma cT4N0M0. What would you recommend first?

A. gastrectomy

B. preoperative chemotherapy

C. preoperative radiotherapy

D. preoperative chemoradiation

13. A 47y otherwise healthy female with gastric adenocarcinoma cT2N2M0. What would you recommend first?

A. gastrectomy

B. preoperative chemotherapy

C. preoperative radiotherapy

D. preoperative chemoradiation

14. Which regimen do you usually use in the neoadjuvant setting?

A. CF(DDP+5FU)

B. ECF(EPI+DDP+5FU)

C. fluoropyrimidine( 5Fu or Capecitabine)

D. oxaplatin based regimen

E. irinotecan based regimen

F. paclitaxel or docetaxel based regimen

G. combination of above or other_______________

15. How long would you usually give neoadjuvant chemtherapy to a patient?

A. <1 m

B. 1-3m

C. >3m

16. A 38y otherwise healthy male with moderately differentiated adenocarcinoma pT4N0M0 underwent gastrectomy 3w ago. What would you recommend?

A. observe without postoperative treatment

B. postoperative chemotherapy

C. postoperative radiotherapy

D. postoperative chemoradiation

17. A 38y otherwise healthy male with moderately differentiated adenocarcinoma pT4N0M0 underwent gastrectomy 3w ago. If you give him adjuvant chemotherapy, what would you recommend?

A. Systemic chemotherpy

B. Intraperitineal chemotherpy

C. Systemic chemotherpy+Intraperitineal chemotherpy

D. Other________________

18. A 57y otherwise healthy male with gastric cancer underwent gastrectomy 3w ago. Moderately differentiated adenocarcinoma invaded muscularis and vascular. There is no metastasis in the 16 removed regional lymph nodes. What would you recommend?

A. observe without postoperative treatment

B. postoperative chemotherapy

C. postoperative radiotherapy

D. postoperative chemoradiation

E. other______________

19. A 42y otherwise healthy female with gastric cancer underwent gastrectomy 3w ago. Poor differentiated adenocarcinoma invaded muscularis. There is no metastasis in the 16 removed regional lymph nodes. What would you recommend?

A. observe without postoperative treatment

B. postoperative chemotherapy

C. postoperative radiotherapy

D. postoperative chemoradiation

E. other______________

20. A 61y otherwise healthy female with gastric cancer underwent gastrectomy 3w ago. Well differentiated adenocarcinoma invaded muscularis. There is no metastasis in the 6 removed regional lymph nodes. What would you recommend?

A. observe without postoperative treatment

B. postoperative chemotherapy

C. postoperative radiotherapy

D. postoperative chemoradiation

E. other______________

21. When would you usually begin adjuvant chemotherapy after a patient undergoes gastrectomy?

A. 1-2w

B. 3-4w

C. 5-6w

D. ≥6w

22. Which regimen do you usually use in the adjuvant setting?(without neoadjuvant chemotherapy)

A. CF(DDP+5FU)

B. ECF(EPI+DDP+5FU)

C. fluoropyrimidine( 5Fu or Capecitabine)

D. oxaplatin based regimen

E. irinotecan based regimen

F. paclitaxel or docetaxel based regime

G. combination of above or other_______________

23. How long would you usually give adjuvant chemtherapy to a patient?

A. 1-3m

B. 4-6 m

C. 7-9 m

D. 10-12 m

E. ≥12 m

F. 3-6 m in the first year, then 2-3m annually for 2-3y

G. other________________

24. How often would you usually recommend a patient to follow-up?

A. Every 4-6m for 3y, then annually

B. Every 4-6m for 5y, then annually

C. Every 2-3m for 3y, then annually

D. Every 2-3m for 5y, then annually

E. other________________

25. Which would you include in the follow-up (multiple answers)?

A. Complete history and physical examination

B. CBC, Hb，platetet

C. hepatic and renal function

D. endoscopy

E. chest CT

F. abdominal CT

G. Vit B12

H. Trace elements and electrolytes

I. other________________

26. Which would you recommend patients as food supplementation(multiple answers)?

A. iron

B. Trace elements and electrolytes

C. Vit B12

D. other_______

27. A 55y female diagnosed with gastric adenocarcinoma and underwent curative resection 4 y ago. She is otherwise healthy, but abdominal CT scan shows solitary liver metastases. Which would you recommend?

A. Systemic chemotherpy

B. liver lesion resection

C.TACE

D. liver lesion resection + systemic chemotherpy

E. other________________

28. A 45y male diagnosed with gastric adenocarcinoma and underwent curative resection 2 y ago. He complains abdominal distension and ultrasound shows large quantity of ascites in which cancer cells are found. His chest CT scan shows no tumor. Which would you recommend?

A. Systemic chemotherpy

B. Intraperitineal chemotherpy

C. Systemic chemotherpy+Intraperitineal chemotherpy

D. Other________________

29. A 45y male diagnosed with gastric cancer and underwent curative resection without chemotherapy or radiotherapy 2 y ago. He is otherwise healthy, but chest CT scan shows lungs metastases. Which would you recommend?

A. CF(DDP+5FU)

B. ECF(EPI+DDP+5FU)

C. fluoropyrimidine( 5Fu or Capecitabine)

D. oxaplatin based regimen

E. irinotecan based regimen

F. paclitaxel or docetaxel based regime

G. combination of above or other_______________

30. A 45y male diagnosed with gastric cancer and underwent curative resection 2 y ago. Chest CT scan shows lungs metastases and disease progressed after 2 cycles of ECF(EPI+DDP+5FU). He is otherwise healthy. Which would you recommend?

A. change regimen and continue chemotherapy

B. Best supportive care

C. Other_________________________

31. A 45y male diagnosed with gastric cancer and underwent curative resection 2 y ago. Chest CT scan shows lungs metastases and disease progressed after 2 cycles of ECF(EPI+DDP+5FU).Then he received 2 cycles of Paclitaxel+DDP chemotherapy. Now there are liver metastases and he is otherwise healthy. Which would you recommend?

A. change regimen and continue chemotherapy

B. Best supportive care

C. Other_________________________

32. A 45y male diagnosed with gastric cancer and underwent curative resection 2 y ago. Chest CT scan shows lungs metastases and disease progressed after 2 cycles of ECF(EPI+DDP+5FU).Then he received 2 cycles of Paclitaxel+DDP chemotherapy. Now there are liver metastases and his PS is 3. Which would you recommend?

A. change regimen and continue chemotherapy

B. Best supportive care

C. Other_________________________

Do you find any question hard to answer?

___________________________________________________________________________

The reason is

___________________________________________________________________________

Do you have any comments on this survey?

_________________________________________________________________________________________________________________________________________________________________________________________________________________________________________________________________________________________________________

We are very appreciated it that you finish the questionnaires!
